# Improving real-world skills in people with intellectual disabilities: an immersive virtual reality intervention

**DOI:** 10.1007/s10055-023-00759-2

**Published:** 2023-04-03

**Authors:** Stefan Carlo Michalski, Nicholas Charles Gallomarino, Ancret Szpak, Kieran William May, Gun Lee, Caroline Ellison, Tobias Loetscher

**Affiliations:** 1 University of South Australia, Adelaide, Australia; 2 The University of Sydney, Sydney, Australia

**Keywords:** Virtual reality, Intellectual disability, Training, Learning, Transfer, Skill generalisation, Adaptive functioning, Cybersickness

## Abstract

Virtual reality (VR) is a promising tool for training life skills in people with intellectual disabilities. However, there is a lack of evidence surrounding the implementation, suitability, and effectiveness of VR training in this population. The present study investigated the effectiveness of VR training for people with intellectual disabilities by assessing (1) their ability to complete basic tasks in VR, (2) real-world transfer and skill generalisation, and (3) the individual characteristics of participants able to benefit from VR training. Thirty-two participants with an intellectual disability of varying severity completed a waste management training intervention in VR that involved sorting 18 items into three bins. Real-world performance was measured at pre-test, post-test, and delayed time points. The number of VR training sessions varied as training ceased when participants met the learning target (≈ 90% correct). A survival analysis assessed training success probability as a function of the number of training sessions with participants split by their level of adaptive functioning (as measured on the Adaptive Behaviour Assessment System Third Edition). The learning target was met by 19 participants (59.4%) within ten sessions (*Mdn* = 8.5, IQR 4–10). Real-world performance significantly improved from pre- to post-test and pre- to delayed test. There was no significant difference from post- to delayed test. Further, there was a significant positive relationship between adaptive functioning and change in the real-world assessment from the pre-test to the post- and delayed tests. VR facilitated the learning of most participants, which led to demonstrations of real-world transfer and skill generalisation. The present study identified a relationship between adaptive functioning and success in VR training. The survival curve may assist in planning future studies and training programs.

## Introduction

People with intellectual disabilities exhibit deficits in both intellectual functioning (e.g. learning, problem-solving, judgement) and adaptive functioning (American Psychiatric Association 2022). Adaptive functioning refers to the conceptual, social, and practical skills required to engage in and undertake everyday activities at the level expected by developmental and sociocultural standards (Balboni et al. 2020; Tassé et al. 2016). Intellectual disability is a broad diagnostic category as it includes individuals with varying levels of impairment. People with severe-to-profound intellectual disabilities typically demonstrate poor adaptive functioning and thus depend on families, caregivers, and paid support for performing everyday activities well into adulthood (Woolf et al. 2010). Finding effective ways for people with intellectual disabilities to learn life skills is crucial for building independence and reducing caregiver burden. Ideally, individuals would practice life skills with caregivers or paid supports, though this is not always feasible given time and resource constraints (Choi et al. 2012; Lindsay and Lamptey 2019; Panerai et al. 2018). There is considerable interest in finding ways to support people with intellectual disabilities to learn independently (de Oliveira Malaquias and Malaquias 2016).

Virtual reality (VR) is gaining interest as a safe, controlled, and repeatable training tool for people with intellectual disabilities (de Oliveira Malaquias and Malaquias 2016; Nabors et al. 2020; Panerai et al. 2018). While many studies have used technology-based interventions for training people with intellectual disabilities, most have used non-immersive virtual environments (e.g. desktop computers) (Kellems et al. 2022; Michalski et al. 2021; Räty et al. 2016; Standen and Brown 2005). More recently, some studies have investigated the use of immersive VR (e.g. head-mounted displays) for training people with intellectual disabilities (Cherix et al. 2020), though few have assessed skill transference to real-world scenarios.

Immersive VR may be an effective way to improve life-skill training approaches for people with intellectual disabilities. Traditional teacher-centred approaches require learners to understand written and spoken language, which is problematic given that many people with intellectual disabilities have impaired communication abilities (Alford et al. 2016; Barker et al. 2013; Klang et al. 2020). Immersive VR may help facilitate experiential or hands-on learning with task-specific benefits (Nabors et al. 2020). For example, physical activities, such as waste management, may benefit from a hands-on approach to learning. In training, users can directly engage in a realistic environment and learn from their experiences with real-time feedback relating to their performance. Though, to what degree VR is an effective tool for training real-world skills in people with intellectual disabilities is yet to be determined.

The potential development of cybersickness is one of the most significant drawbacks of immersive VR exposure (Dennison et al. 2016; Szpak et al. 2020). Encouragingly, two studies exploring the most recent generation of VR headsets for people with intellectual disabilities identified minimal cybersickness and found that most users enjoyed their experience (Michalski et al. 2022; Wang et al. 2021). The studies engaged users in recreational activities and found headset and controller functionality suitable for this population. These findings provide a basis to move from recreational to learning activities, given the low experiences of cybersickness. However, many aspects of virtual reality may contribute to cybersickness experiences, including both software and hardware design components. Thus, it is necessary to assess cybersickness upon development of new virtual reality applications.

The purpose of VR training is to improve skills in the real world. Therefore, it is critical to demonstrate that VR training improves real-world performance (Harris et al. 2020; Michalski et al. 2019b). The ideal VR training tool achieves two outcomes (1) skill transference to the real world and (2) skill generalisation. Rather than only assessing improvement in the virtual training task, it is also crucial to investigate whether training leads to improvement in real-world performance (Abernethy and Wood 2001; Gray 2017). Further, an essential component when interpreting the usefulness of training is to ensure that the participants demonstrate an ability to generalise skills or demonstrate knowledge of learned concepts to new and untrained stimuli.

While the theoretical benefits of VR training for people with intellectual disabilities have been proposed repeatedly (Jeffs 2010), there is a lack of studies demonstrating the effectiveness of training in this population. People with intellectual disabilities present limitations in forming abstract relationships and identifying similarities between categories (Rodrigues et al. 2019). An essential first step is thus to investigate the effectiveness of VR training in a simplistic task. An ideal model for a simple task is waste management, as users will be required to engage various cognitive skills such as learning, memory, and recognition (Tichon 2006). Furthermore, education around everyday activities such as waste management is relevant as it is a life skill that encourages positive behaviour.

As VR training is an emerging area of research, there are unknowns surrounding implementation, given there is a lack of precedent for people with intellectual disabilities. Few studies have considered learning theories to advise the characteristics or methods of their VR training studies. A key consideration in designing studies is the number of provided training sessions. One approach is to pre-determine a certain number of training sessions. However, it may not be clear what that number should be, given the variance in task complexity and capability of the population. An alternative approach is to pre-determine a learning target (Fransson et al. 2020; Hamilton et al. 2021; Lee and Shea 2020). The probability of participants meeting the learning target can then be assessed as a function of the number of training sessions needed to achieve it. Furthermore, whether this probability is modulated by individual factors such as general adaptive functioning can then be investigated.

This study aims to explore the effectiveness of VR training in improving real-world skills in people with intellectual disabilities. The following will be explored. Firstly, the proportion of participant’s able to complete basic tasks in VR. Secondly, it will be assessed whether real-world performance significantly improves following VR training and whether skills generalise to new and untrained stimuli. Thirdly, the relationship between participant’s individual characteristics (adaptive functioning) and real-world performance will be investigated (Miller and Bugnariu 2016). A general adaptive composite (GAC) will be calculated to provide an overall estimate of adaptive functioning inclusive of conceptual skills (communication, functional academics, self-direction), social skills (leisure and social skill areas), and practical skills (community use, home living, health and safety, self-care, work) (Tamm et al. 2022). This study will provide foundational evidence for researchers, educators, and developers in planning research studies and training programs for people with intellectual disabilities.

## Method

### Participants

Forty-five people with a severe-to-profound intellectual disability were recruited from a non-profit organisation in South Australia. Thirteen participants were excluded due to failing the VR tutorial (*n* = 6), VR sickness (*n* = 2), disinterest in trying VR (*n* = 2), external reasons (*n* = 2), and mobility restrictions (*n* = 1). The participants that withdrew due to VR sickness reported mild symptoms of eye strain, nausea, and dizziness, so the researcher opted to discontinue. Thus, 32 participants were included in the analyses. Participant characteristics are reported in Table 1.

**Table 1:** Participant characteristics

Number of subjects	Total	32
	Male	20
	Female	12
Age in years	Mean	38.2
	Standard deviation	16.6
	Range	19–74
Adaptive domain classification per participant (GAC standard score and percentile)*	Low (71–79 GAC, 3–8 percentile)	2
	Extremely low (70 or less GAC, < 2 percentile)	30
Comorbidity (*n*)	Autism spectrum disorder	2
	Cerebral palsy	1
	Down syndrome	17
	Fragile X syndrome	1
Speaks clearly and distinctly**	Is not able	4
	Never (or almost never) when needed	1
	Sometimes when needed	16
	Always (or almost always) when needed	11
Geographical area of residence (*n*)	Adelaide, South Australia	32

Data include included participants only; *Adaptive Behaviour Assessment System Third Edition (ABAS-3) descriptive classifications of GAC (general adaptive composite): high (120 or more), above average (110–119), average (90–109), below average (80–89), low (71–79), extremely low (70 or less); **Indicated by raters on item 8 in the communication section of the ABAS-3

Required sample size was not determined a priori as all of the organisations' clients were invited to participate in the study and we had no clear idea of the effect size to expect. However, a sensitivity power analysis in G*Power (Erdfelder et al. 1996) for detecting improvements in real-world performance (pre- vs post-VR training) was conducted with our sample (*n* = 32) using a paired samples *t* test and an alpha of 0.05. Our sample size provides 80% power to detect an effect of Cohen’s *d* = 0.44. Thus, our sample size is sufficient to reliably detect medium effect sizes (i.e. *d* > 0.44).

### Design

**Fig. 1:**
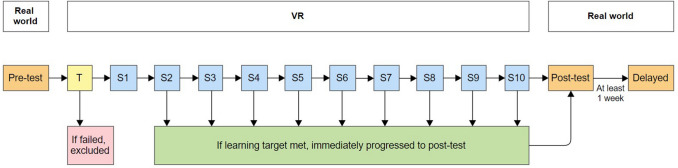
Study design. Participants completed pre-test, post-test, and delayed assessments in the real world. If participants passed the virtual reality (VR) tutorial (T) they moved onto training and completed up to ten VR training sessions (S1–S10). Training ceased early if participants met the learning target (achieving ≈ 90% correct) from session 2 onward

The present study employed a within-subjects design. Real-world performance was measured at pre-test, post-test, and delayed time points. Immediately following the pre-test, participants completed a VR tutorial that involved basic usage of the system to determine the suitability of the intervention. If participants failed the tutorial, they were excluded. If successful, they progressed to VR training. There were a minimum of two and a maximum of ten sessions each. Thus, the number of VR training sessions varied. Training ceased if participants met the learning target by scoring at least 16 out of 18 items correctly in a session (≈ 90%). Participants were required to complete a minimum of two training sessions to ensure they understood the content beyond chance (if successful in session 1). Immediately following the participant's final VR training session, they completed the post-test. The delayed test was scheduled for at least one week after the post-test, see Fig. 1. Further details are explained in the procedure.

### Ethics approval

This study was granted ethics approval from the University of South Australia Human Research Ethics Committee (Protocol No. 202640).

### Materials

#### VR apparatus

The Oculus Quest 1 (developed by Meta Quest; https://www.meta.com/au/quest/) head-mounted display (HMD) was used. The headset has a resolution of 1440 × 1600 per eye at 72 Hz, with approximately 115 degrees of diagonal field of view. Immersive HMDs such as the Quest enable users to view a three-dimensional environment that moves in accordance with the user’s movements in real time. Users can move freely in the physical environment, while their movement is reflected in the virtual environment. The device is capable of inside-out tracking of its position and orientation through four built-in cameras. It is also capable of tracking the positions and orientations of a pair of wireless handheld Oculus Touch controllers. While wearing the HMD, participants used one controller only to interact in the virtual environment. Eyeglasses were worn in the device, if needed.

#### VR tutorial

**Fig. 2:**
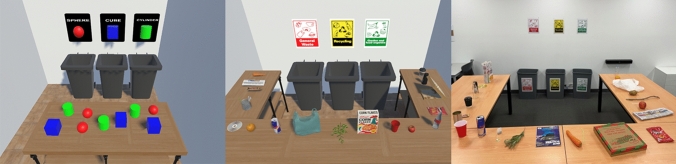
Environments: VR tutorial (left), VR training application (middle), real-world environment (right)

A custom program was built using Unity 3D game engine (developed by Unity Technologies; http://unity3d.com). Participants were required to physically move, grab objects, and move them into the correct bin in the application. Three different objects were used: red sphere, blue cube, and green cylinder. The aim was to move the shapes into the correct bin out of the three options. Objects were moved by holding the trigger on the controller and dropped by releasing the trigger. Participants received no feedback when placing the objects into the bins. See Fig. 2 for a bird’s eye view of the tutorial application.

#### VR training application

Another VR application using Unity 3D game engine was built in which participants were required to physically move, grab objects, and move them into the correct bin. Latency was minimal as the simulator ran as a standalone application on the Quest, there were no performance issues associated with the app, and it maintained 70–72 frames per second. Eighteen everyday items were used (six per bin), see Table 2. There were three options: general waste, recycling, and garden and food organics. Objects were moved by holding the trigger on the controller and dropped by releasing the trigger. When the participant placed an item in the correct bin, feedback appeared (green tick and sound to indicate successful placement), and the item disappeared. When the participant placed an item in an incorrect bin, feedback appeared (red cross and sound to indicate unsuccessful placement), and the item returned to its original location on the table to be re-attempted. When the session was complete, an applauding sound was presented. The sounds used were monoscopic and not spatialised. See Fig. 2 for a birds-eye view of the virtual environments.

**Table 2:** List of items used in virtual reality and the real-world assessment per bin

Items	General waste	Recycling	Garden and organics
In both virtual reality and real-world tasks	CD	Aerosol spray can	Apple
	Chips packet	Cereal box	Carrot
	Chocolate wrapper	Empty tin	Greasy pizza box
	Disposable coffee cup	Glass bottle	Green leaves
	Plastic bag	Newspaper	Green tea bag
	Plastic cup	Soft drink can	Orange
Additional items in the real-world task only	Plastic spoon	Magazine	Potato
	Plastic straw	Plastic water bottle	Tree branch

Bin contents were relevant to South Australian council guidelines

#### Real-world environment

The real-world environment replicated the VR environment, i.e. the table layout was similar, and the signs above the bin were identical. The real-world items were organised in a random order on the table before each session. See Fig. 2 for a birds-eye view of the real-world environment.

#### Items

The items used in the real-world assessment were matched to the VR items used for training in terms of shape, appearance, and size. Six additional items were added in the real-world assessment (two per bin), see Table 2. Thus, 24 items were used (eight per bin), consisting of 18 trained and six untrained items.

### Measures

#### VR performance

VR performance was measured by the percentage of correct items per session. All analyses combined data from the participant’s attempts in both the first and second rounds (see procedure).

#### Real-world performance

The number of items placed in the correct bin was tallied. The scores could range from 0 (no items in correct bins) to 24 (all items in correct bins).

#### Adaptive functioning

The norm-referenced Adaptive Behaviour Assessment System Third Edition (ABAS-3) (Harrison and Oakland 2015) was used to assess adaptive functioning and to classify participants (extremely low, low, below average, average, above average and high). The measure comprises 239 individual behaviour ratings under the following skill areas: communication, community use, functional academics, home living, health and safety, leisure, self-care, self-direction, social, work. Staff who knew the participants well (*n* = 2) completed the adult form, providing behaviour ratings of zero (is not able), one (never or almost never), two (sometimes) or three (always or almost always). The ABAS-3 provides behaviour measures for three adaptive domains: conceptual, social, and practical. A general adaptive composite (GAC) was calculated that is composed of all measured skill areas, thus providing an overall estimate of adaptive behaviour. Higher scores reflect a greater level of adaptive functioning. The ABAS-3 demonstrates good reliability and validity. Correlation coefficients of GAC scores as assessed on the ABAS-3 adult form were used to assess test–retest reliability (0.83) and interrater reliability (0.83) (Harrison and Oakland 2015). Thus, the ABAS-3 has good reliability.

#### Cybersickness

To assess cybersickness, the researcher asked participants whether they felt dizzy or sick. Specifically, the researcher handed the participants a sheet that stated, “I felt dizzy or sick…” Below this statement, there were three response options, “no”, “not sure”, or “yes”. Each option had an emoticon underneath, a smiley face, confused face, and nauseous face, respectively. Participants were required to select an option by pointing to a response on the card or saying the word aloud. The researcher guided the participants through the question to ensure comprehension. If participants could not provide a clear answer, a response was not recorded. If participants reported cybersickness, follow-up questions were asked to gain further insight on their symptoms.

### Procedure

Information sheets were addressed to both participants and their caregivers (if applicable). Informed consent was obtained from participants and a staff member at the organisation. An easy-to-read consent form with pictures was developed to ensure participants had a clear understanding of what was involved in the study. Once informed consent had been obtained, staff completed the ABAS-3.

#### Real-world pre-test

Participants were given brief instructions regarding the typical contents of the three bins. The following was explained: “the general waste bin includes items such as soft plastics and non-reusable items. The recycling bin includes items such as cardboard, bottles, cans, and paper. The garden and food organics bin include items such as fruit and vegetable scraps and things you might find in the garden”. The signs above the bins also had images of typical bin contents. Once participants were familiarised, they were asked to begin placing items in bins, one at a time. The goal of the task was to place all the items into the correct bins. They did not receive any assistance and were instructed to use their best judgement if they were unsure. The real-world assessment was completed once participants had attempted each item.

#### VR tutorial

Following the pre-test, participants underwent the VR tutorial. Participants were given brief instructions regarding the tutorial's aim—move the objects from the table into the correct bin. The researcher also guided participants by showing them the button to grab objects. The researcher assisted with adjusting the headset directly on participants and assuring they could see objects without blurriness. Following the VR tutorial, the researcher assessed participant’s cybersickness.

Participants needed to place at least eight out of nine items in the correct bin to pass the VR tutorial. Participants were excluded from the study if they failed following a maximum of three attempts. The VR tutorial was considered incomplete if, after multiple reminders, any of the following occurred: non-responsive in VR, not following the task instructions, or not clicking the required button at the appropriate time. A researcher remained present to remind and assist participants during the tutorial. Participants that completed the VR tutorial progressed to VR training.

#### VR training

The first VR training session was attempted immediately following the VR tutorial. The researcher provided participants with brief instructions regarding the aim of the task—move the objects from the table into the correct bin.

There were two rounds per session. During the first round, participants were required to place all the items in their correct bin. When items were placed in the correct bin, the item disappeared, and positive feedback appeared. When items were placed incorrectly, negative feedback appeared, and the item returned to its original location on the table to be re-attempted. Once all the items were entered correctly, the second round began. During the second round, only the items placed incorrectly at least once in the first round reappeared. Thus, items placed correctly on the first attempt in the first round did not reappear. The second round served as an opportunity for participants to practice more challenging items. If participants placed an item incorrectly in the second round, it reappeared on the table until placed in the correct bin (same as the first round). The session ended once participants placed all the items into their correct bin in the second round.

There were a maximum of ten and a minimum of two training sessions. Training ceased if participants met the learning target in a session, placing at least 16 out of 18 items correctly (without errors) in the first round. If participants met the learning target, they immediately progressed to the post-test. If participants did not meet the learning target within ten sessions, they progressed to the post-test following their tenth session.

The researcher aimed to schedule a minimum of two sessions with participants per week. However, the time between sessions varied, and COVID-19 restrictions impacted the schedule. If participants were making recurrent errors on the same items while not demonstrating improvement, the researcher ended the session after 15 min. The researcher used a progress sheet with coloured stickers to enable participants to track their progress throughout the experiment and motivate them to complete more sessions.

#### Real-world post-test and delayed test

The real-world assessment completed during the post-test and delayed test was identical to the pre-test. The post-test was completed immediately following the final VR training session. The researcher aimed to complete the delayed test at least seven days after the final VR training session.

## Results

### Descriptives

The VR tutorial was completed with a median time of 1.24 min (Interquartile range 0.9–1.75). Duration of VR training sessions varied as sessions ended once participants had successfully placed all items in their correct bins. On average, sessions lasted 6.69 min (*SD* = 3.11). VR training was completed (first to last session) on an average of 17.7 days (*SD* = 11.6) per participant. The duration of the real-world assessment (minutes) differed at pre-test (*M* = 4.39, *SD* = 1.8), post-test (*M* = 3.03, *SD* = 1.04) and delayed (*M* = 3.2, *SD* = 1.29) time points. The number of days between post-test and delayed test ranged from 7 to 36 days (*M* = 10.4, *SD* = 7.3).

### VR tutorial

Thirty-eight participants attempted the VR tutorial. Six participants did not complete the tutorial: one failed, and five did not finish as they were unable to understand the task and/or confidently navigate the virtual environment. The remaining 32 participants completed the VR tutorial with an average of 8.86 correctly placed objects (*SD* = 0.34).

### VR training

Thirty-two participants completed VR training. Overall, the percentage of correctly placed items significantly increased from the first (*M* = 56.8, *SD* = 18.08) to the last VR training session (*M* = 74.5, *SD* = 24.49) as revealed in a paired *t* test, *t*(31), *p* < 0.001. Cohen’s *d* = 1.22.

The relationship between adaptive functioning and VR training improvement was examined. A Pearson correlation revealed a non-significant relationship between VR training improvements (change score calculated as percentage correct in last VR session minus percentage correct in first VR session) and the general adaptive composite, *r*(30) = 0.27, *p* = 0.067.

**Fig. 3:**
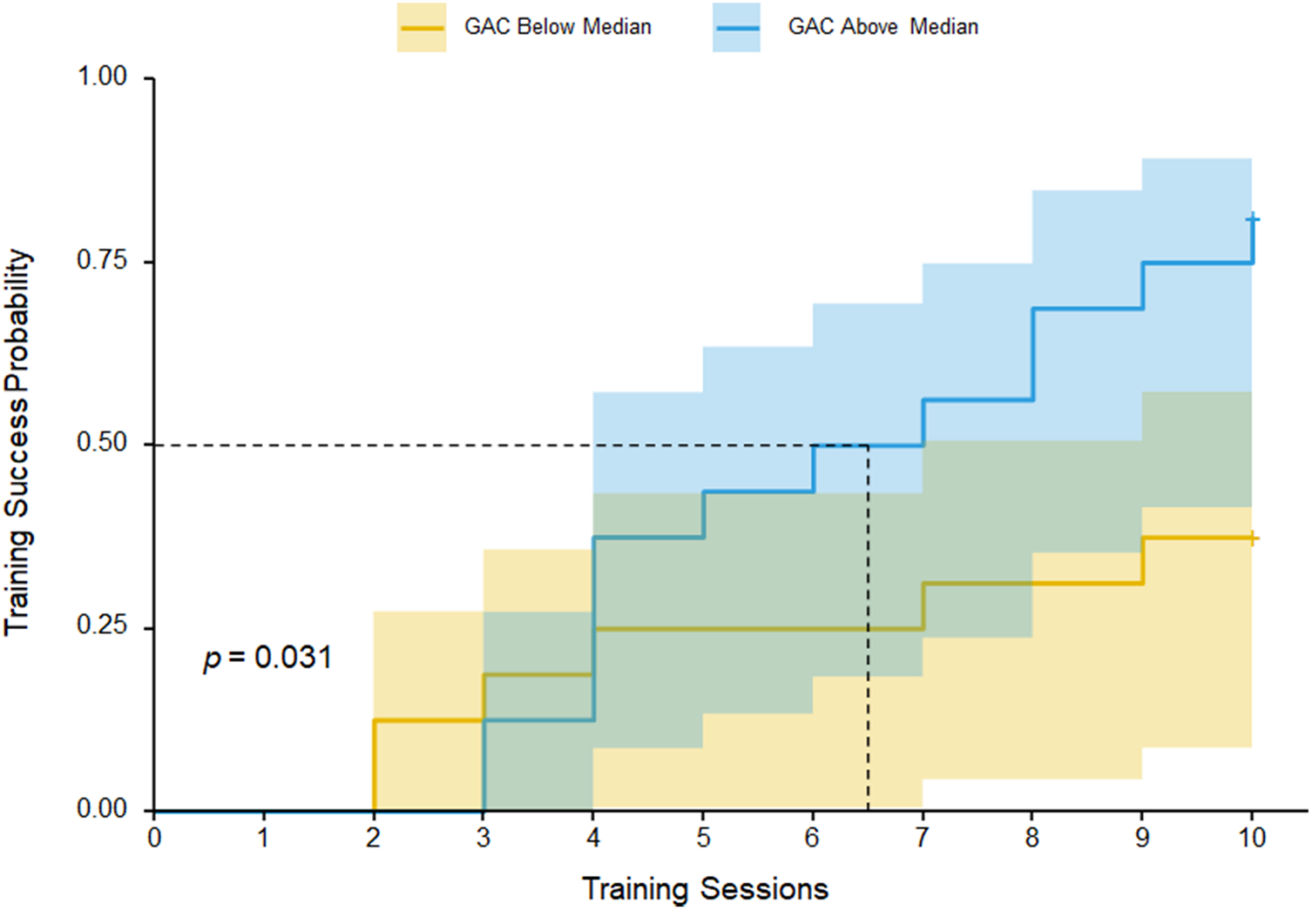
Cumulative training success (proportion of participants meeting the learning target) as a function of the number of training sessions. Participants were split by general adaptive composite (GAC) into above-median (blue line) and below-median (yellow line) groups with 95% confidence intervals. The dotted line represented when 50% of participants met the learning target in the above-median group. Fifty per cent of the below-median group did not meet the learning target within ten sessions; thus, a dotted line is absent. A vertical rise in the curves indicates an event

The learning target was met by 19 participants (59.4%), while 13 participants (40.6%) did not meet it before training ceased. The median number of training sessions was 8.5 (Interquartile range 4–10). A survival analysis using the R survival and survminer packages was conducted to assess training success probability (achieving ≈ 90% correct) as a function of training sessions. Participants were split by the median GAC score (62.5) into an above median and below median group. A censored event indicates a participant did not meet the learning target. The above median GAC group was able to meet learning target in significantly fewer training sessions than the below median GAC group (*p* = 0.031), see Fig. 3. This data includes only the participants who completed the tutorial and progressed to VR training.

### Real-world performance

Real-world performance was assessed at pre-test (*M* = 14.9, *SD* = 3.81), post-test (*M* = 18.1, *SD* = 5.05) and delayed (*M* = 17.5, *SD* = 4.44) time points. A repeated-measures ANOVA was performed to compare the effect of time on real-world performance. Post hoc tests were conducted with Bonferroni–Holm adjustments to correct for multiple comparisons. There was a statistically significant main effect of time points (*F*(31, 2) = 13, *p* < 0.001, *η*_*p*_^2^ = 0.296). A series of paired samples *t* tests were conducted to assess whether there were differences in the real-world assessment over time. Further, differences in trained and untrained items over time were also separately analysed, see Table 3.

**Table 3:** Comparisons of real-world performance for trained and untrained items at the three measuring time points

Stimuli	Test (difference from time 1 to time 2)	Mean difference	*t*(31)	Corrected *p* value	Cohen’s *d*
All items (24)	Pre-post	3.18	3.9	.002*	0.68
	Pre-delayed	2.56	4.22	.003*	0.7
	Post-delayed	− 0.62	− 1.19	.24	0.21
Trained items (18)	Pre-post	2.68	4.15	.003*	0.73
	Pre-delayed	2	3.89	.002*	0.69
	Post-delayed	− 0.68	− 1.64	.112	0.29
Untrained items (6)	Pre-post	0.5	2.18	.074	0.4
	Pre-delayed	0.56	2.87	.021*	0.5
	Post-delayed	0.06	0.26	.79	0.04

Cohen’s *d* effect size interpretation: 0.2 = small effect size; 0.5 medium effect size; 0.8 large effect size. Instances of negative t-values indicate higher scores in time 1 than time 2

The relationship between adaptive functioning and change scores in the real-world assessments was examined. There was a significant positive relationship between GAC and change in the real-world assessment from pre-test to post-test (*r*(30) = 0.379, *p* = 0.032) and from pre-test to delayed (*r*(30) = 0.389, *p* = 0.028). There was no significant relationship between GAC and change from the post-test to delayed (*r*(30) = − 0.139, *p* = 0.447). Separate analyses were also conducted for conceptual, social, and practical scores, see Table 4.

**Table 4:** Correlations of adaptive functioning and change in real-world performance

Adaptive domain	Pre-post	Pre-delayed	Post-delayed
*General adaptive composite*			
*r*	0.379*	0.389*	− 0.139
*p*	0.032	0.028	0.447
*Conceptual*			
*r*	0.377*	0.345	− 0.187
*p*	0.033	0.053	0.304
*Social*			
*r*	0.486**	0.435*	− 0.252
*p*	0.005	0.013	0.165
*Practical*			
*r*	0.307	0.318	− 0.109
*p*	0.087	0.076	0.552

*p < .05, **p < .01

### Cybersickness

Cybersickness was assessed following 250 out of the 270 VR sessions conducted in the study. Data were missing in 20 cases as participants could not provide a clear answer; however, there were no apparent signs of concern. Five participants (15.6%) reported cybersickness symptoms in at least one session throughout the intervention. Symptoms included dizziness and eye strain. Additionally, two participants were excluded from the study for mild cybersickness during the VR tutorial and therefore did not proceed to the VR intervention. In these instances, participants were asked to take a seat until the symptoms subsided. From our observations, the participant’s symptoms were absent within 30 min.

## Discussion

The present study is one of the first to investigate immersive VR training for people with intellectual disabilities using a head-mounted display and handheld controller. Encouragingly, participants performance improved in VR over time. There was a significant increase in the percentage of correctly disposed items from the first to the last VR training session. Crucially, participants were able to demonstrate skill transference following VR training. Real-world performance significantly improved from pre- to post-test, and from pre- to delayed test. There was also no significant difference from post-test to delayed test, suggesting skills were retained up to one week later. The findings in this study are intriguing given the common learning challenges people with intellectual disabilities encounter (Patel et al. 2020). It appears there is value in using VR training for improving real-world skills in people with intellectual disabilities.

Findings from the real-world assessment indicate that participants were able to transfer and generalise their learned skills in a real environment. To investigate skill generalisation, an additional six items were included in the real-world assessment that were untrained, i.e. never appeared in VR. Encouragingly, separate analyses of the six untrained items revealed a similar trend; with small to medium effect sizes for real-world performance from pre- to post-test and pre- to delayed test (although the pre- to post-test was no longer statistically significant (*p* = 0.074) after Bonferroni–Holm corrections for multiple comparisons). For the untrained items, there was no significant difference from post-test to the delayed test, suggesting skills were retained. This finding suggests that participants demonstrated skill generalisation by identifying trends and applying learned principles beyond the immediately trained stimuli. Given few items were used (six), this finding is promising but needs to be confirmed in further studies.

Overall, VR experiences were suitable as participants could engage in the learning task while wearing a headset and using a controller. The VR tutorial was a helpful way to determine the suitability of VR learning before beginning the intervention. Most participants that were excluded during the tutorial struggled with navigating the virtual environment. It is important to note that while six participants failed the tutorial and thus did not proceed to VR training, this does not necessarily mean that VR training is unsuitable for these participants. These participants may have benefitted from a more gradual introduction to VR (Parsons and Mitchell 2002; Tsikinas and Xinogalos 2018). Technological advancements, such as improvements in hand tracking (Buckingham 2021), may also help to reduce the barrier for participants that struggled with controller functionality.

VR experiences were tolerated well as only two participants were excluded due to cybersickness. The researcher’s decision to exclude was considered precautionary as follow-up questioning regarding the severity of symptoms was inconclusive, given the participant's language barriers. Seventeen participants had Down syndrome—a condition with a high occurrence of eye abnormalities (Krinsky-McHale et al. 2014), and thus a likely high predisposition to cybersickness. Nevertheless, most participants exhibited no symptoms of cybersickness. The few participants who did report cybersickness in this study experienced mild and short-lived dizziness and eye strain. This result is encouraging given the wide variety of comorbidities included in the study.

The approach used in the study helped to shed light on the number of training sessions required to observe learning in people with severe-to-profound intellectual disabilities. Instead of pre-determining a set number of training sessions (e.g. 3, 5 or 10), which is common in most VR intervention studies, training ceased once participants met the learning target. This methodology enabled us to conduct a survival analysis to investigate the probability that participants benefited from VR training as a function of the number of training sessions and their individual characteristics. The curve might give other researchers important clues on the number of training sessions needed when planning similar studies.

The survival curve helped identify the participants most likely to benefit from VR training. Participants with higher and lower adaptive functioning (defined as above- and below-median groups) met the learning target on a similar trajectory initially (from sessions 1 to 4). However, from session five onward, the above-median group continues upward, while the below-median group flattens. That is, participants in the above-median group continued to meet the learning target, while the below-median group did not. Notably, only two participants in the below-median group were able to meet the learning target once they were past the halfway mark of the intervention. Levels of adaptive functioning may provide an indication to researchers and educators when considering how much time they should allocate before ceasing or modifying VR training if noticeable improvements have not been made.

Interestingly, people with higher levels of adaptive functioning were more likely to demonstrate skill transference in the real world. Higher adaptive functioning was associated with greater improvement from pre- to post-test and pre- to delayed test in the real-world assessment. These findings suggest that people with a higher level of adaptive functioning were more likely to achieve meaningful improvement following training. However, more research is needed to test this notion, given the effect was not replicated within VR training. We were not able to confirm a significant relationship (*p* = 0.067) between adaptive functioning and improvement in VR training despite the effect size for this correlation being near-moderate in strength (*r* = 0.27). It remains unclear whether the absence of statistical significance could be related to a lack of statistical power for this analysis.

As the GAC comprises three adaptive domains, we explored the relationship of each to improvements in real-world performance to determine which domains are most relevant. A significant relationship was found between pre- to post-test improvements on conceptual scores, and pre- to post-test improvements on social scores. In VR training, participants were tasked to recognise trends, follow directions, and use memory and recognition skills. Therefore, it is unsurprising that conceptual skills were relevant to training success. Furthermore, during training, social skills were required as participants needed to demonstrate listening skills to follow the instructions and rules of the task. Interestingly, a non-significant relationship was found between pre- to post-test improvements and practical scores. That is, better practical skills were not associated with better training outcomes. The practical skills required to complete this task were relatively simple (only clicking one button to grab items and move naturally) and were mastered by all participants passing the tutorial task. This suggests that participants did not need exceptional practical skills to improve on the task. It is conceivable that levels of practical skills could be more relevant for VR training applications that require more complex interactions.

There is scope to amplify the positive effects found in this study. The present study used a simple learning paradigm that involved learning by repetition. Participants were exposed to all the stimuli and were asked to place each item into the correct bin, repeating only incorrect items. A way to improve the VR intervention may have been to design the intervention using a learning model supported by a strong evidence base. VR offers a unique ability to implement principles of evidence-based practice that are difficult to structure into real-world training (Michalski et al. 2019a, b; van Vonderen 2004; Zahabi and Abdul Razak 2020). Perhaps it would have been more effective to individualise training to each participant, for example, by adjusting the difficulty of a task relative to success in training (i.e. adaptive training) or repeating more challenging content at increasing intervals (i.e. spaced repetition) (Gray 2017; Standen et al. 2020; Zahabi and Abdul Razak 2020). Furthermore, investigating VR training in a between-groups design may enable investigation into the effectiveness of VR in comparison with other interventions and help control for practice effects. A larger sample would also be beneficial to reduce potential risk of Type II error.

Overall, the findings from this study support the notion that skills learned in VR transfer to the real world. The findings of VR learning are intriguing given the common learning challenges people with intellectual disabilities encounter. While encouraging, the results do not indicate that VR training is equal to or better than real-world training or other technology forms (e.g. iPad or Cave Automatic Virtual Environment) as this was beyond the scope of the study. Indeed, VR will likely not replace the quality of human interaction, but it may provide value to complement teachings and be useful in situations where there are limited resources for training in real life (Hamilton et al. 2021; Lee et al. 2019; Walker et al. 2016). An important next step will be to compare VR training with other training forms and an integration of evidence-based learning models into training. Further, as the present study investigated people with intellectual disabilities, future research is needed to determine if the results can be generalised to groups with different neuropsychological profiles.

## Data Availability

The datasets generated during and/or analysed during the current study are available from the corresponding author on reasonable request.
